# Dissecting Endoplasmic Reticulum Unfolded Protein Response (UPR^ER^) in Managing Clandestine Modus Operandi of Alzheimer’s Disease

**DOI:** 10.3389/fnagi.2018.00030

**Published:** 2018-02-06

**Authors:** Safikur Rahman, Ayyagari Archana, Arif Tasleem Jan, Rinki Minakshi

**Affiliations:** ^1^Department of Medical Biotechnology, Yeungnam University, Gyeongsan, South Korea; ^2^Department of Microbiology, Swami Shraddhanand College, University of Delhi, New Delhi, India; ^3^School of Biosciences and Biotechnology, Baba Ghulam Shah Badshah University, Rajouri, India; ^4^Institute of Home Economics, University of Delhi, New Delhi, India

**Keywords:** Alzheimer disease, neurodegenerative diseases, endoplasmic reticulum stress (ER), aging, UPR (unfolded protein response)

## Abstract

Alzheimer’s disease (AD), a neurodegenerative disorder, is most common cause of dementia witnessed among aged people. The pathophysiology of AD develops as a consequence of neurofibrillary tangle formation which consists of hyperphosphorylated microtubule associated tau protein and senile plaques of amyloid-β (Aβ) peptide in specific brain regions that result in synaptic loss and neuronal death. The feeble buffering capacity of endoplasmic reticulum (ER) proteostasis in AD is evident through alteration in unfolded protein response (UPR), where UPR markers express invariably in AD patient’s brain samples. Aging weakens UPR^ER^ causing neuropathology and memory loss in AD. This review highlights molecular signatures of UPR^ER^ and its key molecular alliance that are affected in aging leading to the development of intriguing neuropathologies in AD. We present a summary of recent studies reporting usage of small molecules as inhibitors or activators of UPR^ER^ sensors/effectors in AD that showcase avenues for therapeutic interventions.

## Introduction

Alzheimer’s disease (AD), the most common form of dementia faced by more than 40 million people worldwide, significantly affect morbidity and mortality in aged people (Alzheimer’s Association, 2016; Fiest et al., 2016; Scheltens et al., 2016; Cass, 2017). The most vulnerable group falling as target is above 65 years, which puts aging as the crucial risk factor associated with development of the disease (Alzheimer’s Association, 2016; Fiest et al., 2016; Scheltens et al., 2016; Cass, 2017). AD is a progressively neurodegenerative disorder, characterized by cognitive alterations and behavioral changes that owe to synaptic impairment and loss of neurons (Alzheimer’s Association, 2016; Scheltens et al., 2016). Mutations in genes encoding APP (amyloid precursor protein), presenilin 1 and 2 (PS1 and PS2 respectively), as well as ε4 allele of Apolipoprotein E are reported to be linked to rare familial and early development of AD (Selkoe, 2001a,b; Scheltens et al., 2016). AD leads to the formation of neurofibrillary tangles having hyperphosphorylated microtubule associated tau protein and senile plaques of amyloid-β (Aβ) peptide in specific brain regions, result in brain inflammation, astrogliosis and microglial proliferation (Citron, 2002; Selkoe, 2004a,b; Cleary et al., 2005; Haass and Selkoe, 2007; Atwood and Bowen, 2015; Minter et al., 2016; Sami et al., 2017). Gradual accumulation of Aβ peptide attributed to β- and γ-secretases action on the APP, results in synaptic loss and neuronal death (Chyung et al., 2005; Tatarnikova et al., 2015).

The expression pattern of neurodegenerative pathologies shows distinct molecular signatures, such as misfolded Aβ aggregation and tau protein hyperphosphorylation in the brain (Jiang et al., 2010; Atwood and Bowen, 2015; Sami et al., 2017). How this load of protein aggregates disrupt the neuronal function is still a mystery to medical science? In this review, we have tried to focus on the role of ER stress and the ensuing unfolded protein response (UPR^ER^) imposed on the neuronal cell due to misfolded protein aggregates. Also, we have discussed various therapeutic interventions targeting the molecules involved in UPR pathways aiming at averting the neuropathologies of AD.

## ER Stress and UPR^ER^

Adversities in the endoplasmic reticulum (ER) microenvironment like nutrient deprivation, changes in redox potential, calcium homeostasis, hypoxia and accumulation of unfolded/misfolded protein triggers the UPR^ER^ (Schroder and Kaufman, 2005; Moneim, 2015). UPR^ER^ is a highly conserved signaling cascade in all eukaryotes involved in the cellular homeostasis (Ellgaard and Helenius, 2003; Mori, 2009; Walter and Ron, 2011) through transcriptional remodeling of ER proteostasis pathways (Lee et al., 2003; Yamamoto et al., 2007; Shoulders et al., 2013; Genereux et al., 2015). The ER lumen harbors various molecular chaperones like the Glucose Regulated Protein 78 kDa (GRP78) that are recruited to misfolded nascent peptides for aiding in their proper folding (Bertolotti et al., 2000; Shen et al., 2002). A plethora of studies have reported UPR^ER^ upregulation in the brain samples of Alzheimer’s patients (Hamos et al., 1991; Hoozemans et al., 2005, 2009).

**Figure 1:**
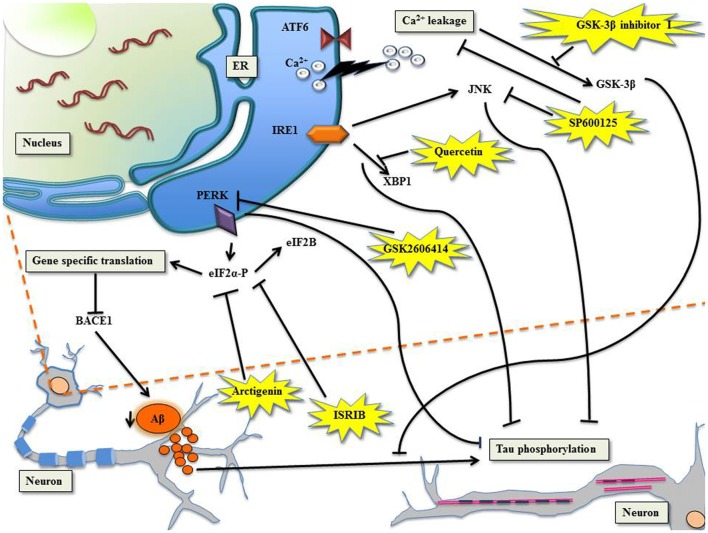
Targeting unfolded protein response (UPR) to manage Alzheimer’s disease (AD) with specific molecules. *PERK/eIF2α*: the phosphorylation of eIF2α shuts down global translation in the cell but for gene-specific translation upregulation of mRNA with internal ribosome entry site (IRES), for example β-site APP cleaving enzyme-1 (BACE1), the β-secretase enzyme (Ohno, 2014). Arctigenin, targets eIF2α-P thereby downregulating BACE1, consequently protects neurons from amyloid-β (Aβ) toxicity. ISRIB, affects eIF2B leading to inhibition of eIF2α-P, which comprehensively restores protein translation and hence enhances long term memory. PERK can be directly inhibited by GSK2606414, leading to halt in tau phosphorylation. Ca^2+^ leakage induced activation of glycogen synthase kinase-3β (GSK-3β) can be checked by GSK-3β inhibitor I, which prevents Aβ induced phosphorylation of tau. *IRE1/XBP1*: Quercetin, activates endoribonuclease activity of IRE1 inhibiting tau hyperphosphorylation. The c-Jun N-terminal kinase (JNK) inhibitor, SP600125, inhibits Ca^2+^ leakage and inhibits Aβ-induced c-Jun phosphorylation.

The UPR^ER^ embodies a complex network comprised of three stress-responsive transmembrane proteins, Protein Kinase RNA like ER kinase (PERK), Inositol Requiring Element 1 (IRE1) and Activating Transcription Factor 6 (ATF6; Figure 1; Schroder and Kaufman, 2005; Walter and Ron, 2011; Minakshi et al., 2017; Rahman et al., 2017). PERK, a type 1 transmembrane kinase protein, gets trans-autophosphorylated and homodimerized after activation, thereby promoting phosphorylation of serine residues on cytoplasmic eIF2α (eukaryotic initiation factor 2 alpha; Harding et al., 1999; Bertolotti et al., 2000; Ma et al., 2002; Marciniak et al., 2006). Despite the general translational halt induced by the phosphorylated eIF2α (eIF2α-P), certain specific mRNAs bearing internal ribosome entry site (IRES), like the Activating Transcription Factor 4 (ATF4) mRNAs continues to be translated (Harding et al., 2000a; Baumeister et al., 2005). ATF4 regulates genes for various foldases, chaperones, regulatory proteins of the redox and autophagy, cholesterol metabolism etc. (Harding et al., 2003; Fusakio et al., 2016). CCAAT enhancer-binding (C/EBP) protein homologous protein (CHOP) is also a direct target of ATF4 and represents the pro-apoptotic component of the UPR^ER^ (Han et al., 2013). In a study, wild type mice subjected to tunicamycin injection showed higher degrees of apoptosis in their renal epithelium as compared to CHOP knockout mice (Marciniak et al., 2004; Onuki et al., 2004). PERK also induces the activation of another transcription factor nuclear factor (erythroid derived 2)-like 2 (Nrf2) independent of eIF2α, which regulates the antioxidant response (Cullinan et al., 2003; Cullinan and Diehl, 2004).

IRE1 is the most evolutionarily conserved ER stress transducer (Tirasophon et al., 1998), which upon activation, undergoes dimerization and trans-autophosphorylation, leading to the activation of its cytosolic endoribonuclease activity that splices a 26-nucleotide intron from the mRNA encoding transcription factor X box binding protein 1 (XBP1) forming XBP1(S) (Yoshida et al., 2001, 2003). The XBP1(S) upregulates genes involved in ER protein maturation and ER-associated degradation (ERAD; Lee et al., 2003; Acosta-Alvear et al., 2007). Cells lacking XBP1 are more sensitive to hypoxia-induced apoptosis (Romero-Ramirez et al., 2004). Upon activation, IRE1 also activates c-Jun N-terminal kinase (JNK) through tumor necrosis factor receptor-associated factor 2 (TRAF2); Zeng et al., 2015). IRE1-mediated JNK activation has been demonstrated to trigger autophagy under ER-stress (Urano et al., 2000).

ATF6 is a type II transmembrane protein, with a basic leucine zipper (bZIP) domain (Yoshida et al., 1998). During the imposed stress, luminal domain of ATF6 loses its association with GRP78, triggering the translocation of ATF6 into the Golgi apparatus where two intramembrane Golgi specific proteases, site 1 protease (S1P) and site 2 protease (S2P), process it. The N-terminal cleaved product p50ATF6 of full length ATF6 (p90ATF), then acts as a transcription factor, which upregulates several genes, including GRP78, Protein Disulfide Isomerase (PDI), XBP1 and CHOP (Haze et al., 1999; Walter and Ron, 2011).

## UPR^ER^ in Alzheimer’s Disease

In neuronal pathophysiology, the activation of UPR^ER^ can have paradoxical affects. During stress condition, activation of UPR^ER^ could reactivate proteostasis; thereby rescuing the neurons by escalating the rate of protein folding through molecular chaperones, or may trigger neurodegeneration and neuronal collapse through the expression of apoptotic markers.

Evidences support the presence of abundant hyperphosphorylated tau protein and ER stress markers in the neurons of the cortex in postmortem brain samples of AD patients (Scheper and Hoozemans, 2015). It is presumed that ER stress is a cell death mechanism triggered by Aβ, and is linked to changes in ER calcium homeostasis (Cornejo and Hetz, 2013). Under the influence of Aβ imposed ER stress, Ca^2+^ leaching from ER is taken up by mitochondria leading to activation of apoptotic death of neurons (Fonseca et al., 2013). The presenilins are responsible for passive ER Ca^2+^ outflow. Documents support that aging neurons fail to maintain tight Ca^2+^ homeostasis across plasma membrane and ER (Supnet and Bezprozvanny, 2010). Such effects paved the way for “calcium hypothesis of brain aging and AD” (Khachaturian, 1989). Rise in prolonged imbalanced Ca^2+^ invites ROS accumulation and mitochondrial dysfunction resulting in neuronal death (Supnet and Bezprozvanny, 2010). ER stress may display binary role in AD, firstly modulating the production kinetics of amyloid plaques and secondly altering the cognitive functions in a distinct way (Halliday and Mallucci, 2015). Neurons of AD patients were also characterized by GRP78 induction in temporal cortex and hippocampus and phosphorylation of PERK (p-PERK; Hoozemans et al., 2005).

Active protein synthesis is a hallmark feature of synaptic plasticity and consolidation of memory (Costa-Mattioli et al., 2009). PERK signaling and protein translation control was linked to the cognitive impairment observed in AD models (Devi and Ohno, 2013, 2014). Impairment of cognitive functions due to the reduction in synaptic protein synthesis is displayed during increased phosphorylation of eIF2α (Costa-Mattioli et al., 2005, 2009; Jiang et al., 2010). Mitigating the expression of PERK improves cognitive function and synaptic plasticity in an AD model (Devi and Ohno, 2014). Moreover, targeting other eIF2α kinases like General Control Nonderepressible-2 (GCN2) and dsRNA-dependent protein kinase R (PKR) was also witnessed not only to improve learning and memory processes (Devi and Ohno, 2013), but also reduced inflammation (Lourenco et al., 2013). These results significantly indicate that genetic manipulation of PERK improved cognitive ability of cells to survive under stress conditions induced by Aβ deposition.

The activation of UPR^ER^ in early stages of AD could be protective through activation of autophagy. However, sustained UPR^ER^ activation may be detrimental to the neurons (Hoozemans et al., 2005; Nijholt et al., 2011). The expression of XBP1 in *Drosophila* where the AD-associated Aβ peptide was expressed in neurons, led to reduced neurotoxicity, supporting the cytoprotective role of XBP1 (Casas-Tinto et al., 2011). In *Caenorhabditis elegans* (*C. elegans*) models expressing aggregation-prone mutant tau variants, XBP-1 was identified to be playing a similar protective role (Kraemer et al., 2006; Loewen and Feany, 2010). However, reports also suggest that IRE1 interacts with PS1 leading to activation of proapoptotic signaling by JNK (Shoji et al., 2000). The JNK3 (member of JNK family) localized in brain, is highly expressed in brain tissue and cerebrospinal fluid sample from AD patients (Gourmaud et al., 2015) and the activation of JNK3 exacerbates stress perpetuating AD pathology (Yoon and Jo, 2012).

## Aging, UPR^ER^ and Alzheimer’s Disease

Aging is the single most important risk factor for AD. Decline in the UPR^ER^ with advancing age marked by the oxidative damage of ER chaperones, leads to disempowering of protein folding capacity (Rabek et al., 2003; Nuss et al., 2008). Studies report that the levels of GRP78 were low in murine cortex, in rat hippocampus, cortex, cerebellum, as well as in a multitude of organs (Paz Gavilán et al., 2006; Hussain and Ramaiah, 2007; Naidoo et al., 2008). Transcription of PERK mRNA were lowered in the aging rat hippocampus, while an increment was reported in the expression of growth arrest and DNA damage protein 34 (GADD34), because it escapes the effect of eIF2α-P translational inhibition (Paz Gavilán et al., 2006). Studies on *C. elegans* revealed that the activation of IRE1 branch of the UPR^ER^ diminishes during the fertile period of adulthood, manifesting in lowered immunity against ER stress (Taylor and Dillin, 2013). The implication of IRE1/XBP1 tier in aging was proven in *C. elegans* where IRE1 defect reduced life span (Chen et al., 2009).

## Mitochondria, Oxidative Stress and Alzheimer’s Disease

**Figure 2:**
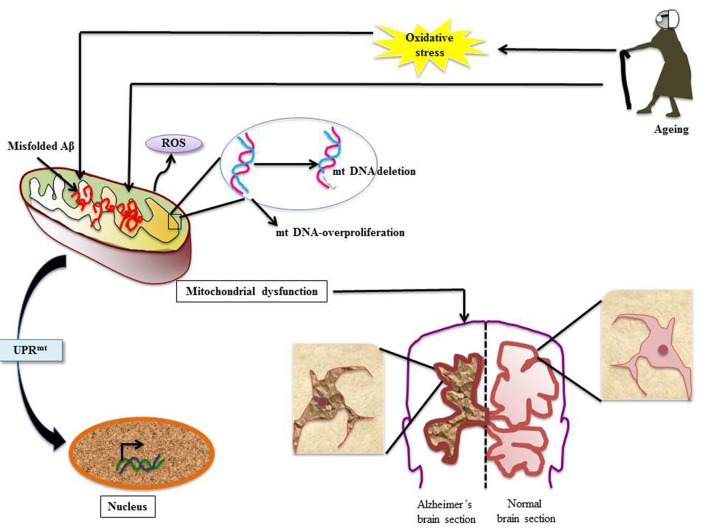
Mitochondrial dysfunction in AD: various stress insults like aging and oxidative stress disrupt client protein folding in mitochondria thereby invoking mitochondrial UPR (UPR^mt^). Numerous events line up; there is mitochondrial DNA (mtDNA) proliferation and deletion, misfolded Aβ overload and ROS generation. This leads to the condition of mitochondrial dysfunction and to rescue the ailing cell UPR^mt^ is stimulated. The effect of mitochondrial dysfunction leads to development of neuropathologies associated with AD (Aliev et al., 2008, 2013; Onyango et al., 2016).

Under the imposed stress, apart from UPR^ER^ coming to the rescue, the herald of mitochondrial UPR (UPR^mt^) ensuing after accumulation of unfolded peptide load is well documented. The pathway focuses on invigorating folding and degradation of misfolded peptides in mitochondrial matrix through the execution of retrograde transcriptional activation (Arnould et al., 2015). AD being a multifactorial malady, the accumulation of Aβ not only affects ER but also mitochondria. There are accumulating evidences, which support deposition of Aβ in mitochondrial matrix disrupting signaling of the organelle thereby leading to neurodegeneration (Kawamata and Manfredi, 2017). Impairment in the production and functionalities of metabolic enzymes preferentially of TCA cycle disturbs energy metabolism of the brain. Mitochondrial dysfunction causes depletion of cellular ATP pool and enhanced ROS production, which is well implicated in the pathogenesis of AD (Swerdlow et al., 2014; Hoekstra et al., 2016). Besides, impairment of mitochondrial turnover and function in brain, aging potentiates oxidative stress, leading to significant decrease in the cytochrome C oxidase activity that is associated with rise in oxygen radicals in different regions of postmortem AD brain (Figure 2; Hirai et al., 2001; Mosconi et al., 2007; Krishnan et al., 2012). A strong correlation of the cognitive decline with increase in oxidative stress is observed in AD patients (Revel et al., 2015). Incidence of aberrant Aβ processing ensues after the oxidation of mitochondrial DNA (mtDNA) under stressful circumstances (Volgyi et al., 2015).

Aberrations in mtDNA have been well studied in AD. In an elegant study by Aliev et al. (2013) mtDNA-proliferation and deletion has been reported in AD tissues. Furthermore, the report also illustrates abnormal mitochondrial function in damaged hippocampal neurons in human AD as well as transgenic AD models. In another study using *in situ* hybridization, Aliev et al. (2008) detected a 5 kB deletion in mtDNA under oxidative stress in abnormal neurons. Such mitochondrial anomalies were also reported to help in AD pathogenesis in Aβ transgenic mice (Aliev et al., 2008).

In a study proving the existence of interlink between mitochondrial dysfunction and AD, the pharmacological/genetic targeting of mitochondrial translation process not only increased life span of GMC101 (model of Aβ proteotoxicity), but also showed reduction in beta-amyloid aggregation in worms and transgenic mouse models of AD (Sorrentino et al., 2017). Treatment of the mitochondrial division inhibitor-1 (mdiv-1) that inhibits mitochondrial fragmentation, thereby rescuing mitochondrial distribution, improves mitochondrial function in CRND8 (AD mouse model) neurons (Reddy et al., 2017; Wang et al., 2017). Treatment with mdivi-1 also causes a decrease in extracellular amyloid deposition and Aβ1–42/Aβ1–40 ratio (Wang et al., 2017). Additionally, SIRT-3, a sirtuin localized to inner mitochondrial membrane, has been found associated with enhancement in the levels of glutathione (Onyango et al., 2002; Someya et al., 2010). As downregulation of SIRT-3 was found to be having a retrograde effect on p53 mediated mitochondrial and neuronal damage in AD, its modulation by therapeutics was found to ameliorate mitochondrial pathology and neurodegeneration in AD (Lee et al., 2018).

## Derangement of Glucose Metabolism in Alzheimer’s Disease: The Fallible UPR^ER^

Among the many observed hallmarks of AD, positron emission tomography (PET) revealed a deranged glucose metabolism in brain regions. Aging registers diminished brain glucose utilization that surges in AD (Ivançević et al., 2000). Various reports suggest that UPR^ER^ is linked to abnormal glucose metabolism and insulin resistance (Hetz et al., 2015). Type 2 diabetes mellitus (T2DM) has been mechanistically linked to AD pathogenesis, where higher insulin resistance poses a greater risk of AD with reduced glucose uptake in the brain as well as memory loss (Willette et al., 2015; Wijesekara et al., 2017). In addition, there is decline in key neuronal glucose transporters, GLUT1 and GLUT3, as shown in AD mouse models (Ding et al., 2013). The exact molecular mechanism underlying the effect of glucose uptake in AD model is not completely understood, but evidences suggest a close link between AD and insulin signaling. Apart from controlling glucose metabolism, insulin also regulates neural development with respect to learning and memory (Ying et al., 2017).

The lowering in glucose concentration due to lack of active transporters (GLUT1 and GLUT3) instates mitigating effect on hexosamine pathway (HBP), due to which O-GlcNAcylation is compromised with hyperphosphorylation on tau protein (Liu et al., 2009). XBP1(S) is shown to directly target the rate limiting enzyme of HBP, glutamine fructose-6-phosphate aminotransferase (GFAT1; Wang et al., 2014), as XBP1(S) transgenics showed rise in O-GlcNAcylation (Wang et al., 2014). The situation of insulin resistance established in aging has also been shown to increase HBP flux (Einstein et al., 2008). A gain-of-function mutation in GFAT1 of *C. elegans* showed significant induction of ERAD and autophagy favoring longevity (Denzel et al., 2014).

Protein aggregation is a consequence of AD which is a result of abnormal proteostasis in the cell (Kaushik and Cuervo, 2015). An increase in the UPR^ER^ driven protein homeostasis was observed with the overexpression of GLUT1 as this promoted downregulation of expression of GRP78. GRP78, being the negative regulator of the UPR^ER^, binds ATF6 and IRE1 thereby continuing them in an inactive state. One interesting study showed that flies (with increased glucose transport) when fed with the drug metformin showed mitigated levels of GRP78 with ensuing gain in lifespan, additionally the expression of GLUT1 and its association with the beginning of UPR^ER^ exerted neuroprotective effect (Niccoli et al., 2016).

## Targeting UPR^ER^ to Manage AD

The involvement of ER stress and hence the UPR^ER^ in neuropathologies exposes the molecules of the pathway as attractive targets for therapeutic interventions. Here, we have compiled reports from studies that have targeted molecules of UPR^ER^ for managing the deterioration caused by AD (Figure 1).

### eIF2α and PERK in AD

There are accumulating evidences that support increased phosphorylation of PERK and eIF2α in AD (Chang et al., 2002; Page et al., 2006; Kim et al., 2007). The processing of highly expressed single-pass transmembrane protein in brain, the amyloid precursor protein, leads to the generation of neurotoxic Aβ during neuropathogenesis. Reports suggest that the secretase β-site APP cleaving enzyme-1 (BACE1), increases APP cleavage as a result of eIF2α phosphorylation leading to the production of Aβ in neurons (O’Connor et al., 2008). The PERK tier of UPR when suppressed leads to the alleviation of synaptic plasticity and memory loss in AD (Ma et al., 2013). The administration of arctigenin, a bioactive product from *Arctium lappa* (L.), has been known to inhibit BACE1 translation through dephosphorylation of eIF2α-P (Zhu et al., 2013). The phosphorylation of eIF2α is central to integrated stress response (ISR) that modulates UPR (Harding et al., 2000b) and formation of memory proteins (Costa-Mattioli et al., 2005). ISR inhibitor (ISRIB) interferes with ISR by affecting eIF2B activity whose competitive inhibitor is eIF2α-P (Krishnamoorthy et al., 2001; Sekine et al., 2015; Bogorad et al., 2017). This comprehensively reverses the effect of eIF2α-P, which resulted in the restoration of translation and hence long term memory enhancement in rodents (Sidrauski et al., 2013, 2015). The genetic deletion of eIF2 kinases, PERK, GCN2 and dsRNA-dependent protein kinase (PKR) ameliorate synaptic plasticity and memory in AD models (Ma et al., 2013). The transient translational halt induced by PERK-P/eIF2α-P was challenged by GSK2606414, a PERK inhibitor, because of which tau phosphorylation could be checked, resulting in the amelioration of neurodegeneration (Axten et al., 2012; Radford et al., 2015). The development of AD manifested by Aβ accumulation forces tau hyper phosphorylation in sync with increased activity of glycogen synthase kinase-3β (GSK-3β) in the cortical neurons (Takashima et al., 1993, 1996; Tomidokoro et al., 2001; De Felice et al., 2008; Resende et al., 2008). Resende et al. (2008) showed that Aβ oligomers cause ER stress linked calcium leakage which in turn leads to GSK-3β activation, the later when inhibited by GSK-3β inhibitor I, led to the prevention of Aβ induced phosphorylation of tau.

### IRE1/XBP1 in AD

The advantageous effects of XBP1 on memory was proven in neural-specific XBP1 knockout mice featuring impaired learning and synaptic plasticity deficit, where injections of adeno-associated viruses delivered XBP1(S) resulted in establishing long-term hippocampus memory (Martínez et al., 2016). In accordance with this finding, another study reinforced the neuro-protective role of XBP1 in AD mice (Casas-Tinto et al., 2011; Cisse et al., 2017). Nonetheless, a flavonol, called quercetin, activated endoribonuclease activity of IRE1 and inhibited tau hyperphosphorylation (de Boer et al., 2006; Suganthy et al., 2016). In cases of familial AD, deletions or mutations in presenilin genes accentuate ER Ca^2+^ leakage. The JNK inhibitor, SP600125, when challenged in PS1/PS2 double knockout mouse embryonic fibroblast, caused inhibition of Ca^2+^ leakage (Das et al., 2012). The neuroinflammation exhibited in AD through tau phosphorylation mediated by the kinase activity of JNK was inhibited by SP600125, consequently inhibiting Aβ-induced c-Jun phosphorylation (Vukic et al., 2009; Zhou et al., 2015).

## Future Directions and Concluding Remarks

ER, being a central organelle in nerve cells, coordinates with the cellular homeostasis by managing translation/modification of proteins and Ca^2+^ equilibrium, thereby maintains the proper signaling in brain. The disruption in neuronal physiology is quite evident in age-related AD where ER dysfunctions are prominently expressed in the form of imbalance in proteostasis. Advancements in studies based on AD models have clearly shown how we can intervene the molecular pillars of UPR^ER^ and its associated signaling cascades to manage neurodegeneration in age-related AD. The present review is an attempt to revise functional relevance of the studies conducted in the field of management of age-related AD through therapeutic interventions on the UPR^ER^ pathway and its associate’s molecules. Studies reinforce that the strategies where intervening the molecules, which are involved in transposing effects of aging on neurodegeneration, will cause reduction in probability of AD pathology. The manifestation of ER proteostasis is a direct indication of healthy nervous system. Progression in AD witnesses glucose hypo-metabolism in brain, reduction in glucose transporters in neurons and endothelial cells of blood brain barrier in direct proportion with the amount of neurofibrillary tangles. Type 2 diabetics with higher insulin resistance are at a greater risk of AD. Recent reports elucidate that managing UPR^ER^ can exert neuroprotective effect in AD (Smith and Mallucci, 2016). Additionally, as evidenced in the study by Sorrentino et al. (2017), the recapitulation of mitochondrial function through activation of UPR^mt^ can impede plaque formation. Aliev et al., also demonstrated link between cancer and AD where mtDNA over-proliferation and deletion induces cell cycle dysregulation prompting oncogenic pathway (Aliev et al., 2013). We have supporting literature that underpins the reversal of AD pathology by anticancer drugs (Cramer et al., 2012). Aiming at therapeutic intervention, the ailing mitochondria can be challenged with specific antioxidants like MitoQ, acetyl-L-carnitine and R-alpha lipoic acid to alleviate AD (Aliev et al., 2011; Volgyi et al., 2015). One remarkable study on astrocytes underpins the protective role of conditioned medium of human mesenchymal stem cells (CM-hMSCA) sourced from adipose tissue against neuropathologies (Baez-Jurado et al., 2017). The state of astrocyte mitochondrial dysfunction has been proven to be a start point for neuronal death (Baez et al., 2016). Pharmacological targeting of astrocytes has been proposed to be a potential way in therapeutics of AD (Baez et al., 2016). A transcriptomic analysis in astrocytes has put forward a conglomeration of various algorithms for strategic approaches in therapeutics of neuropathologies (Barreto et al., 2017).

We still need extensive and efficient model systems where the molecular intricacies of weakened UPR^ER^ in aging-induced neuropathology in AD can be ventured upon, so that pharmacological as well as genetic tools could underscore the significance of UPR^ER^ as well as UPR^mt^ in aged brain.

## Author Contributions

SR and RM conceived the idea. SR, ATJ, AA and RM contributed to writing of the manuscript.

## Conflict of Interest Statement

The authors declare that the research was conducted in the absence of any commercial or financial relationships that could be construed as a potential conflict of interest.
